# Diagnostic Accuracy of Acoustic Radiation Force Impulse (ARFI) in Diagnosis of Liver Fibrosis among Egyptian Patients with Chronic HCV Infection

**DOI:** 10.3889/oamjms.2016.064

**Published:** 2016-06-08

**Authors:** Yasser A. Elhosary, Saleh M. Saleh, Wafaa M. Ezzat, Dirk-André Clevert

**Affiliations:** 1 *Department of Internal Medicine, National Research Center, Cairo, Egypt*; 2 *Department of Internal Medicine, National Liver Institute, Menoufia, Egypt*; 3 *Department of Clinical Radiology, Munich University Hospital, Munich, Germany*

**Keywords:** HCV, ARFI, liver fibrosis, diagnostic accuracy

## Abstract

**BACKGROUND:**

Acoustic radiation force impulse (ARFI) evaluates liver stiffness non-invasively and was invented recently. This technique can easily and accurately assess the degree of liver fibrosis in clinical practice.

**AIM:**

The aim of this study was to detect the diagnostic performance of ARFI elastography in the staging of fibrosis in some Egyptian patients with chronic HCV infection.

**PATIENTS AND METHODS:**

One hundred ninety patients with chronic HCV infection; 142 men and 48 women were enrolled in the study. They underwent liver biopsy examination for liver fibrosis detection. All demographic; clinical and biochemical data were recoded. ARFI examination was done for all subjects to detect liver stiffness measurement in relation to liver fibrosis detected by pathological examination of liver biopsies.

**RESULTS:**

Medians of liver stiffness measurement by shear wave velocity showed a significant increase as a grade of liver fibrosis increases (p ≤ 0.0001, highly significant). Liver stiffness was directly correlated to age, AST; ALT; INR and liver steatosis (p values were: 0.009; 0.0001; 0.013; 0.006 and 0.04 respectively, significant). On the other hand, liver stiffness was inversely correlated to albumin; prothrombin concentration and platelets (p values were: 0.0001; 0.001, and 0.0001, respectively, significant). We found that shear wave velocity can predict F1; F2; F3 and F4 at cut-off values: 1.22; 1.32; 1.44 and 1.8 respectively.

**CONCLUSION:**

ARFI is a diagnostic noninvasive promising technique for liver fibrosis diagnosis among Egyptian patients with chronic HCV infection.

## Introduction

The incidence of chronic hepatitis C has been increasing gradually and is a potentially life-threatening problem especially among Egyptians. Sustained and repeated inflammatory stimuli can cause a stress repair response in the body, leading to the accumulation of a large number of extracellular matrixes in the liver tissue, leading to fibrosis [1]. Progression of fibrosis can cause cirrhosis or even liver cancer. Therefore, early detection is important for the prevention and control of liver fibrosis and cirrhosis. Liver biopsy is the main diagnostic method for liver fibrosis. However, it is an invasive method and associated complications limit its safety [2].

Recently, various non-invasive techniques for liver fibrosis assessment have been developed, including serum biomarkers and elastography techniques. Transient elastography (TE), real-time elastography (RTE), acoustic radiation force impulse imaging (ARFI) are the most frequent elastography techniques [3, 4].

ARFI evaluates liver stiffness non-invasively and was invented recently. This technique can easily and accurately assess the degree of liver fibrosis in clinical practice [5-7]. Fierbinteanu-Braticevici et al [8] reported that ARFI elastography showed very good accuracy in assessing all stages of liver fibrosis. In addition, a meta-analysis by Nierhoff et al [9] also demonstrated good diagnostic accuracy.

Nowadays, early and continuous detection of liver fibrosis has become increasingly important in order to make therapeutic decisions, determine prognosis and to follow-up disease progression. Hepatic fibrogenesis is a dynamic process reflecting an imbalanced extracellular matrix turnover. Evaluating the evolution of fibrosis over time can be therefore more important than a “once only” diagnosis. Recent evidence suggesting that liver fibrosis can be reversible [10, 11], further emphasizes the importance to monitor fibrosis over time, other than simply diagnosing its presence and staging its severity. Accurate, reproducible and easily applied methods are therefore required for the assessment of hepatic fibrosis

The aim of this study was to detect the diagnostic performance of ARFI elastography in the staging of fibrosis in some Egyptian patients with chronic HCV infection.

## Patients and Methods

### Patients

The study was conducted on 190 patients with chronic HCV infection; 142 men and 48 women with documented chronic HCV infection, recruited from Agouza Liver Center and National Research Center, Cairo. Chronic HCV diagnosis was based on elevated serum transaminase levels for at least six months and positive HCV antibody by the second-generation enzyme-linked immunosorbent assay and confirmed by detection of circulating HCV RNA using polymerase chain reaction (PCR). A liver biopsy specimen was taken from every patient. The current study was approved by ethical committee in the national research center. An informed consent was signed by every patient before enrollment in the study. Demographic data; clinical data; biochemical investigations; results of abdominal ultrasonography; histopathological examination of liver biopsies and data of ARFI examination were recoded for every enrolled patient.

We excluded any patients with history renal disorder, the recent history of cardiovascular disease or patients with hepatitis B infection or human immunodeficiency virus infection, autoimmune or metabolic liver diseases; abnormal coagulation profiles that preclude liver biopsies; international normalized ratio (INR) > 1.5, prothrombin time > 50 seconds and platelet count < 50,000/mL. Relative and absolute contraindications to liver biopsy, e.g. biliary ductal dilatation, ascites, and the presence of hepatocellular carcinoma were also excluded.

### Histopathological Examination of Liver Biopsies

Ultrasound-guided percutaneous liver biopsy specimens were taken from the patients and examined by two different pathologists, experienced in liver histology, who were unaware of the laboratory results or clinical diagnosis. Only specimens with the inter-observer agreement of stage of hepatic fibrosis were included in the study. METAVIR scoring system was used for staging hepatic fibrosis. Every biopsy specimen was staged on a scale of F0 to F4 (F0 = no fibrosis, F1 = portal fibrosis without septa, F2 = few septa, F3 = numerous septa without cirrhosis, and F4 = cirrhosis) [12, 13].

### Acoustic radiation force impulse elastography

Acoustic radiation force impulse elastography was performed for all subjects within two months of the date of liver biopsy with a Siemens Acuson S3000 Virtual Touch ultrasound system (Siemens AG, Erlangen, Germany) with a 6CI transducer. The principle underlying ARFI elastography is that sharing of the examined tissue induces a strain in the tissues. An acoustic “push” pulse is automatically produced by the ultrasound probe and directed to the side of a region of interest (ROI), which is where the speed of the shear wave is measured. This ROI has a predefined size, provided by the system (10 mm long and 5 mm wide).

**Figure 1:**
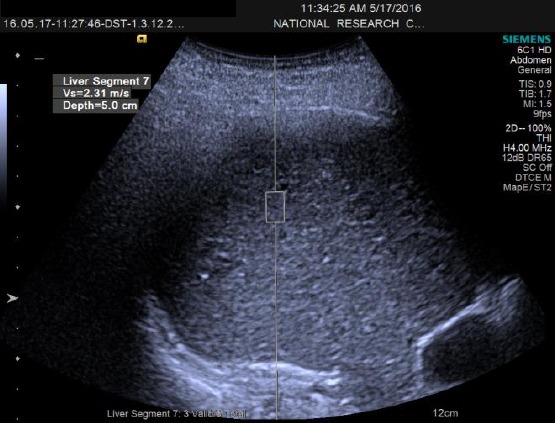
Ultrasonographic picture of ARFI examination for liver fibrosis

**Figure 2:**
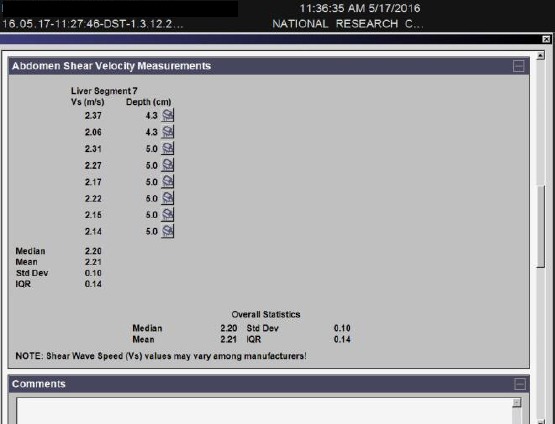
Output report of ARFI examination wit means, median and standard deviation

The acoustic “push” pulse generates shear waves that propagate into the tissue, perpendicular to the “push” axis. Detection waves are also generated by the transducer to measure the propagation speed of these shear waves, which increases with fibrosis severity [14]. The speed of the shear waves, measured in meters per second, as well as measurement depth, is displayed by the system. For each patient, 10 valid ARFI measurements were performed under fasting conditions, with the patient in supine position with the right arm in maximum abduction, by the intercostal approach in the right liver lobe, 1–2 cm under the liver capsule. Minimal scanning pressure was applied by the operator; the patient was asked to stop normal breathing for a moment to minimize breathing motion. The mean of 8-10 valid measurements was calculated and considered indicative of the severity of fibrosis (Fig. 1, Fig. 2). The operators were blinded to any clinical or elastographic data.

### Statistical analysis

Data are expressed as median or mean ± SD or median and range where appropriate. Categorical data are described as the frequency of the subjects with a specific characteristic. Chi-square test or Fisher’s exact test was used for comparing categorical data and Student’s t-test, Mann-Whitney-U-test, or Kruskal-Wallis test, when appropriate, was used for comparing continuous variables. The sensitivity-to-specificity relationship of each noninvasive diagnostic test was assessed by using receiver operating characteristic curves. Area under the receiver operating characteristic curve and the 95% confidence interval were calculated for detection of histologic fibrosis stage 2 or higher disease (F ≥ 2), detection of histologic fibrosis stage 3 or higher disease (F ≥ 3), and detection of cirrhosis (F4) in the entire study population. P ≤ .05 was considered indicative of a significant difference. Statistical analysis was performed using SPSS software version 12.0 (SPSS Inc., Chicago, IL, USA).

## Results

Base line features of the studied patients were present in Table 1. One hundred ninety patients with chronic HCV infection who underwent liver biopsies for pathological examination were enrolled in the current study from May 2014 to May 2015. They were 142 men and 48 women with mean age of 53.74 ± 12.05.

**Table 1:** Patients’ characteristics

	Variables	HCV patients N = 190
Age in years	Mean ± SD	53.74 ± 12.05
Sex	Male No (%)	142 (74.7)
	Female No (%)	48 (25.3)
PH of Schisto:	Negative No (%)	63 (33.2)
	Positive No (%)	127 (66.8)
Viral load IU/mL	Median (range)	114000 (0- 33400000)
AST IU/L	Median (range)	44.50 (1.97-283)
ALT IU/L	Median (range)	38.00 (8.00-270)
Albumin gm/dl	Median (range)	3.90 (1.40-4.01)
Total bilirubin mg/dl	Median (range)	1.20 (0.20-4.5)
Direct bilirubin mg/dl	Median (range)	0.50 (0.00-3.9)
P C %	Median (range)	79.00 (31-199)
INR	Median (range)	1.20 (0.8-3.9)
GGT IU/L	Median (range)	31.00 (1.3-271)
Platelets ×10^3^	Median (range)	130.50 (0.95-512.00)
AFP ng/ml	Median (range)	6.50 (0.20-7900)
CHOL mg/dl	Mean ± SD	167.69 ± 40.36
TRG mg/dl	Mean ± SD	114.85 ± 39.30
Histopathological examination Steatosis % median(range)		9 (4-17)
Fibrosis:		
F 1 No (%)		25 (13.2)
F 2 No (%)		28 (14.7)
F 3 No (%)		29 (15.3)
F 4 No (%)		108 (56.8)
Activity:		
A 0 No (%)		8 (4.2)
A 1 No (%)		64 (33.7)
A 2 No (%)		100 (52.7)
A 3 No (%)		18 (9.4)

PH of Schisto: past history of Schistosomiasis; AST: aspartate transaminase; ALT: alanine transaminase; PC%: prothrombin concentration; INR: international normalized ratio; GGT: Gamma-glutamyl transpeptidase; AFP: α-fetoprotein; CHOL: cholesterol; TRG: triglycerides.

**Figure 3:**
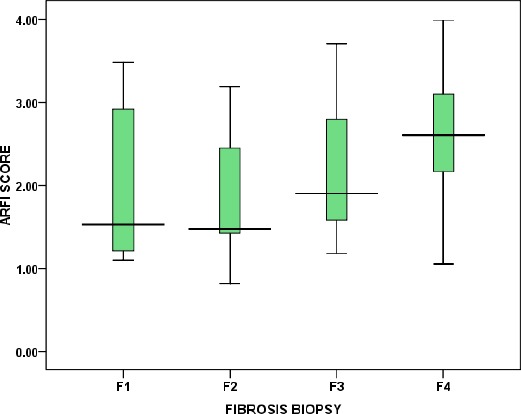
Box Plot diagram for distribution of liver stiffness measurement by ARFI among different grades of liver fibrosis

The frequency of past history of Schistosomiasis was 66.8%. Histopathological examination of liver biopsies revealed that distribution of liver fibrosis grades: F1; F2; F3; F4 was 13.2%; 14.7%; 15.3% and 56.8% respectively. Medians of liver stiffness measurement by sheer wave velocity showed a significant gradual increase as a grade of liver fibrosis increases (p ≤ 0.0001, highly significant) as shown in Table 2 and Fig. 3.

**Table 2:** Distribution of liver stiffness measurements by ARFI among different grades of liver fibrosis

Grades of fibrosis	Shear wave velocity m/sec Median	Shear wave velocity m/sec Range	Χ^2^	P value
F1	1.20	1.10 - 1.40	26.460	0.0001*
F2	1.43	1.26 - 1.62		
F3	1.90	1.43 - 2.80		
F4	2.70	1.52 - 3.90		

* : p is highly significant.

Analyzing correlation of liver stiffness measurement by shear wave velocity to the studied parameters revealed that liver stiffness was directly correlated to age, AST; ALT; INR and liver steatosis (p values were: 0.009; 0.0001; 0.013; 0.006 and 0.04 respectively, significant).

On the other hand, liver stiffness was inversely correlated to albumin; prothrombin concentration and platelets (p values were: 0.0001; 0.001 and 0.0001, respectively, significant) as shown in Table 3.

**Table 3:** Correlation of liver stiffness measurements by ARFI with different studied variables among the studied patients

Variables	Correlation Coefficient R	Coefficient of determination R2.	P value
Age	0.175**	0.031	0.009
AST	0.295**	0.087	0.000
ALT	0.165*	0.027	0.013
Albumin	-0.329**	0.108	0.000
Total bilirubin	0.127	0.016	0.057
Direct bilirubin	0.062	0.004	0.354
P C	-0.224**	0.050	0.001
INR	0.183**	0.033	0.006
GGT	0.036	0.001	0.593
Platelets	-0.300**	0.09	0.000
Viral load	-0.036-	0.001	0.596
AFP	0.110	0.012	0.099
CHOL	-0.086	0.007	0.200
TRG	-0.089	0.008	0.186
Liver steatosis	0.141*	0.020	0.042

AST: aspartate transaminase; ALT: alanine transaminase; PC%: prothrombin concentration; INR: international normalized ratio; GGT: Gamma-glutamyl transpeptidase; AFP: α-fetoprotein; CHOL: cholesterol; TRG: triglycerides;

* : p is significant;

** : p is highly significant.

Multiple regression (assessed by standardized B coefficients) analysis to find an independent predictor for an increase of liver stiffness revealed that platelet count is the independent factor for increasing liver stiffness measurement by ARFI (B = -0.006, P = 0.048, significant). There was no any significant impact of sex or past history of Schistosomiasis on liver stiffness measurements (p values were: 0.812 and 0.262 respectively, insignificant).

**Figure 4:**
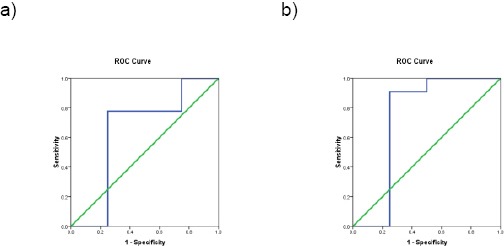
a) ROC curve for diagnosis of liver fibrosis grade 1 by ARFI; b) ROC curve for diagnosis of liver fibrosis grade 2 by ARFI

As regards diagnostic accuracy of ARFI technique for liver fibrosis detection, we found that shear wave velocity can predict F1 at a cut-off value 1.22 with 67.6% sensitivity and 75% specificity. A cut off value1.32 can predict F2 with 75.0 % sensitivity and 90.9 % specificity. A cut-off value 1.44 can predict F3 with 96.6% sensitivity and 75% specificity. A cut-off value 1.8 can predict F4 with 95.7 % sensitivity and 100% specificity Table 4 and Fig. 4.

**Table 4:** Diagnostic performance of ARFI for liver fibrosis diagnosis

	Shear wave velocity m/sec Cut off value	AUR	Specificity	Sensitivity	PPV	NPV
F1	1.22	0.639	75%	67.6%	87.50%	60%
F2	1.32	0.727	90.9 %	75.0 %	90.9 %	75.0 %
F3	1.44	0.905	75%	96.6%	96.6%	100%
F4	1.80	0.989	100%	95.7 %	100%	40%

AUR: area under curve; PPV: positive predictive value; NPV: negative predictive value.

## Discussion

Early detection of liver fibrosis is very important for proper management of chronic liver disease patients, especially in our country. To best of our knowledge, this study is the first to detect accuracy of ARFI as a non-invasive diagnostic technique for liver fibrosis among Egyptian patients. All enrolled patients were recruited from Agouza Liver Center and outpatient’s clinics of National Research Center., these patients come from all governorates of Egypt seeking medical advice, thus, we think our patient’s sample represents patients all over the country.

In the current study, we found that ARFI elastography can predict different grades of liver fibrosis with reasonable sensitivity and specificity. The area under the curve was highest for F ≥ 3 and F = 4. Thus, the ability to diagnose late fibrosis (F ≥ 3) and cirrhosis (F = 4) is higher than that for early fibrosis (F ≥ 1 and F ≥ 2). Our findings agree with previous studies, a meta-analysis of nine studies [15-23] showed that ARFI imaging had excellent diagnostic accuracy for the staging of liver fibrosis in various chronic liver diseases; compared with liver biopsy, ARFI was highly accurate in the diagnosis of fibrosis stage F ≥ 3 (AUROC = 0.91) and for the diagnosis of liver cirrhosis (AUROC = 0.93) [24]. Moreover, an international multicenter study of 911 HCV mono-infected patients found that ARFI was highly accurate in the diagnosis of fibrosis stage F ≥ 3 (AUROC = 0.83) [25].

Spore and his colleagues reported that in daily practice, seldom more than one noninvasive electrographic method for liver fibrosis assessment is available at any center. Thus, the hepatologist must use the most cost-effective one. According to their results, ARFI elastography could be this method as it has a significantly higher rate of reliable liver stiffness (LS) measurements, compared with transient elastography (TE). Also, as previously noted, most of the published studies [26-31] have reported similar accuracy for ARFI and TE in the evaluation of liver fibrosis. ARFI has another advantage compared with TE: It can be integrated into a standard ultrasound system (which can also be used for standard ultrasound evaluation, contrast-enhanced ultrasound, and/or Doppler examinations).

According to Rizzo and his colleagues [32], their study included 139 patients with chronic HCV infection, ARFI was more accurate than TE for the noninvasive staging of both significant (≥ F2) (AUROC: 0.86 vs. 0.78, P = 0.024) and severe (F3-F4) fibrosis (AUROC: 0.94 vs. 0.83, P = 0.02). Importantly, no cases of invalid measurements were recorded vs. 6.5% of unreliable results in patients undergoing TE (P = 0.029). Moreover, in contrast to TE, liver steatosis does not seem to influence ARFI [33]. Another advantage is that it can be easily incorporated into a modified U/S machine. Further validation including inter- and intra-operator reproducibility is required before ARFI can be used in routine clinical practice.

Lupsor, and his colleagues [34] reported that the cut-off values (m/s) for fibrosis stages were: 1.19 (F ≥ 1), 1.34 (F ≥ 2), 1.61 (F ≥ 3) and 2.00 (F4). The study was performed in 112 patients. When they compared ARFI and TE, the areas under the receiver operating characteristic curves (AUROC) were 0.709 vs. 0.902, P = 0.006 (F ≥ 1), 0.851 vs. 0.941, P = 0.022 (F ≥ 2), 0.869 vs. 0.926, P = 0.153 (F ≥ 3) and 0.911 vs. 0.945, P = 0.331 (F4). Sporea, and his colleagues [10] concluded that the cut-off values for predicting the stages of fibrosis were 1.19 m/s for F ≥ 1 (based on Metavir staging system), 1.21 m/s, (F ≥ 2), 1.58 m/s (F ≥ 3) and 1.82 m/s (F = 4). This was conducted in 247 patients with hepatitis C.

Our findings with that of previous studies contribute to confirming the accuracy of ARFI as a noninvasive promising device for liver fibrosis and cirrhosis. From a practical point of view, management of early fibrosis (F1 and F2) is different from that of late fibrosis (F3) or cirrhosis (F4). Most of these studies reported higher sensitivities and specificities for F3 and F4. So that the ability of ARFI can discriminate late fibrosis (F3) and cirrhosis (F4) from early fibrosis (F1 and F2) increase its feasibility as a noninvasive diagnostic tool for liver fibrosis.

ARFI elastography was properly correlated with biochemical markers for hepatitis as it was directly correlated to AST; ALT; INR while it was inversely correlated to albumin; prothrombin concentration and platelets. Moreover, ARFI elastography increased gradually with the advance of live fibrosis. This is a more added evidence for ARFI as a diagnostic technique for liver fibrosis.

Hepatic fibro genesis is the corner stone of progression of any chronic liver disease. So that, we think it’s time to pay more efforts in the field of detection of liver fibrosis. We search for an accurate; easy; cheap and can be used the frequent method to help us not only in diagnosing liver fibrosis but we can follow up our patients to detect progression or even regression of fibrosis especially among patients who receive antiviral treatment for chronic viral infection. Although Egypt has the highest prevalence of HCV infection, we think that with continuous health promotion and new antiviral treatments, the incidence of chronic HCV infection among Egyptian will be decreased in the near future. But there is another big health problem that threatens Egyptian livers which are a nonalcoholic fatty liver disease (NAFLD). We previously searched for NAFLD among some Egyptian healthy subjects; we found that NAFLD was detected in 47 (65.3%) children and in 52 (62.7%) adults [35]. Fatty liver disease patients in our country usually consider that NAFLD is a normally associated condition with obesity and overweight cases. They do not know the progression of NAFLD into nonalcoholic steatohepatitis (NASH); liver cirrhosis or even hepatocellular carcinoma. These patients cannot agree for their liver to be biopsied for pathological examination. Thus, use of noninvasive accurately, a diagnostic device for liver fibrosis is mandatory for patients and doctors.

For the past 50 years, liver biopsy has been considered to be the gold standard for staging of liver fibrosis. However, many recent studies clearly highlight several crucial drawbacks of liver biopsy, including variable accessibility, high cost, sampling errors and inaccuracy due to inter-and intra-observer variability of pathologic interpretations [36]. In addition, there is a small but important risk of liver biopsy-associated morbidity and mortality, with pain and hypotension as the most frequent complications and intra peritoneal bleeding and injury to the biliary system as the most serious complications. Studies reveal that the risk for hospitalization after liver biopsy is 1-5%, the risk for severe complications is 0.57%, and mortality rates vary from 0.009% to 0.12% [37]. Because of these reasons, some patients may opt to forgo liver biopsy and may not know the stage of their liver disease with important prognostic implications. The liver biopsy is not a golden standard method for liver fibrosis any more, but it could be considered the best available standard method. The activating validity of noninvasive techniques for liver fibrosis detection will help us to observe the liver continuously. Moreover, paving the way for therapeutic intervention aiming to reverse liver fibrosis or at least stop its progression.

In conclusion, acoustic radiation force impulse elastography is a feasible method for assessment of liver fibrosis among Egyptian patients with chronic HCV infection.

**List of abbreviations**
ARFI: Acoustic Radiation Force Impulse
HCV: hepatitis C virus infection
AST: aspartate transaminase
ALT: alanine transaminase
INR: international normalized ratio
TE: Transient elastography
RTE: real-time elastography
ROI: region of interest
AUROC: area under receiving operating curve
